# Supplementary material for: Modeling glioblastoma heterogeneity as a dynamic network of cell states
Source: Mol Syst Biol. 2021 Sep 16;17(9):e10105. doi: 10.15252/msb.202010105 (PMC8444284; doi:10.15252/msb.202010105)
Supplement: Supplementary file 5 — Source Data for Figure 3 [file MSB-17-e10105-s001.zip › Figure3A_sourcedata/GSEA_3065/hallmarks_state6.GseaPreranked.1623416308702/HALLMARK_TGF_BETA_SIGNALING.html]

Details for gene set HALLMARK\_TGF\_BETA\_SIGNALING[GSEA]

|  || Dataset | state6 |
| Phenotype | NoPhenotypeAvailable |
| Upregulated in class | na\_pos |
| GeneSet | HALLMARK\_TGF\_BETA\_SIGNALING |
| Enrichment Score (ES) | 0.36769193 |
| Normalized Enrichment Score (NES) | 1.3379083 |
| Nominal p-value | 0.06937799 |
| FDR q-value | 0.1659363 |
| FWER p-Value | 0.655 |
Table: GSEA Results Summary

  

Fig 1: Enrichment plot: HALLMARK\_TGF\_BETA\_SIGNALING      
 Profile of the Running ES Score & Positions of GeneSet Members on the Rank Ordered List

  

| PROBE | GENE SYMBOL | GENE\_TITLE | RANK IN GENE LIST | RANK METRIC SCORE | RUNNING ES | CORE ENRICHMENT || 1 | RAB31 |  |  | 23 | 0.284 | 0.1089 | Yes |
| 2 | KLF10 |  |  | 234 | 0.166 | 0.1500 | Yes |
| 3 | WWTR1 |  |  | 241 | 0.164 | 0.2136 | Yes |
| 4 | HIPK2 |  |  | 445 | 0.128 | 0.2409 | Yes |
| 5 | MAP3K7 |  |  | 694 | 0.106 | 0.2544 | Yes |
| 6 | PPP1R15A |  |  | 968 | 0.087 | 0.2575 | Yes |
| 7 | ID2 |  |  | 1058 | 0.082 | 0.2797 | Yes |
| 8 | ID3 |  |  | 1329 | 0.070 | 0.2763 | Yes |
| 9 | CTNNB1 |  |  | 1461 | 0.066 | 0.2871 | Yes |
| 10 | HDAC1 |  |  | 1468 | 0.066 | 0.3122 | Yes |
| 11 | TJP1 |  |  | 1659 | 0.058 | 0.3134 | Yes |
| 12 | SMURF2 |  |  | 1693 | 0.057 | 0.3320 | Yes |
| 13 | CDKN1C |  |  | 1788 | 0.054 | 0.3424 | Yes |
| 14 | RHOA |  |  | 1836 | 0.052 | 0.3576 | Yes |
| 15 | SKIL |  |  | 1921 | 0.050 | 0.3677 | Yes |
| 16 | BMPR1A |  |  | 2099 | 0.045 | 0.3651 | No |
| 17 | PMEPA1 |  |  | 2388 | 0.036 | 0.3465 | No |
| 18 | ID1 |  |  | 2451 | 0.035 | 0.3530 | No |
| 19 | NCOR2 |  |  | 2497 | 0.033 | 0.3610 | No |
| 20 | FNTA |  |  | 2552 | 0.032 | 0.3674 | No |
| 21 | UBE2D3 |  |  | 3038 | 0.021 | 0.3203 | No |
| 22 | FKBP1A |  |  | 3098 | 0.020 | 0.3214 | No |
| 23 | PPM1A |  |  | 3563 | 0.010 | 0.2723 | No |
| 24 | ARID4B |  |  | 3592 | 0.009 | 0.2728 | No |
| 25 | TGIF1 |  |  | 3672 | 0.007 | 0.2667 | No |
| 26 | SERPINE1 |  |  | 3811 | 0.004 | 0.2527 | No |
| 27 | JUNB |  |  | 4044 | -0.000 | 0.2262 | No |
| 28 | SPTBN1 |  |  | 4188 | -0.003 | 0.2110 | No |
| 29 | SMAD3 |  |  | 4246 | -0.004 | 0.2061 | No |
| 30 | TRIM33 |  |  | 4753 | -0.013 | 0.1536 | No |
| 31 | FURIN |  |  | 4962 | -0.017 | 0.1367 | No |
| 32 | SKI |  |  | 5202 | -0.022 | 0.1181 | No |
| 33 | APC |  |  | 5342 | -0.025 | 0.1121 | No |
| 34 | SMAD1 |  |  | 5360 | -0.026 | 0.1202 | No |
| 35 | CDK9 |  |  | 5484 | -0.028 | 0.1172 | No |
| 36 | THBS1 |  |  | 5670 | -0.032 | 0.1086 | No |
| 37 | SMURF1 |  |  | 6212 | -0.043 | 0.0636 | No |
| 38 | PPP1CA |  |  | 6444 | -0.048 | 0.0562 | No |
| 39 | TGFB1 |  |  | 6712 | -0.056 | 0.0478 | No |
| 40 | IFNGR2 |  |  | 6997 | -0.065 | 0.0408 | No |
| 41 | TGFBR1 |  |  | 7219 | -0.071 | 0.0436 | No |
| 42 | BMP2 |  |  | 7299 | -0.074 | 0.0638 | No |
| 43 | SLC20A1 |  |  | 7392 | -0.078 | 0.0838 | No |
| 44 | XIAP |  |  | 7394 | -0.078 | 0.1142 | No |
| 45 | BMPR2 |  |  | 8138 | -0.117 | 0.0752 | No |
Table: GSEA details [plain text format]

  

Fig 2: HALLMARK\_TGF\_BETA\_SIGNALING: Random ES distribution      
 Gene set null distribution of ES for **HALLMARK\_TGF\_BETA\_SIGNALING**
